# Exogenous jasmonic acid and salicylic acid enhance selenium uptake and mitigate cadmium accumulation in pak choi (*Brassica chinensis* L.) grown in selenium-rich, high-cadmium soil

**DOI:** 10.3389/fpls.2025.1619522

**Published:** 2025-08-19

**Authors:** Jin-Ping Chen, Jie Qin, Ying Xing, Qing Liao, Li-Ping Pan, Cheng-Cheng Zeng, Yong-Xian Liu

**Affiliations:** ^1^ Agricultural Resources and Environment Research Institute, Guangxi Academy of Agricultural Sciences, Nanning, China; ^2^ Guangxi Key Laboratory of Arable Land Conservation, Guangxi Academy of Agricultural Sciences, Nanning, China; ^3^ Flower Research Institute, Guangxi Academy of Agricultural Sciences, Nanning, China

**Keywords:** selenium biofortification, cadmium mitigation, high-cadmium soil, metal chelation compounds, antioxidant enzyme

## Abstract

Plant hormones are known to regulate the uptake and distribution of mineral elements, including heavy metals, in crops. This study evaluated the effects of exogenous jasmonic acid (JA) and salicylic acid (SA), applied individually or in combination, on selenium (Se) enrichment and cadmium (Cd) mitigation in pak choi (*Brassica chinensis* L.) cultivated in Se-rich and high-Cd soils. Hormone treatments significantly increased shoot Se content by 33.7%-62.3% compared to the control, with the highest level Se accumulation observed under combined application of 50 μmol·L^-1^ JA and 50 μmol·L^-1^ SA. Cd accumulation in shoots decreased by 11.7%-29.3% in JA-containing treatments, with the same combined producing the lowest shoot Cd levels. JA alone increased root Cd content, while SA treatments reduced it. Individual hormone treatments elevated root levels of phytochelatins (PCs), glutathione (GSH), and metallo-thioneins (MTs), while the combined treatment future increased PCs and GSH, but decreased MTs and non-protein thiols (NPTs). Antioxidant enzyme activities (SOD, CAT, POD), chlorophyll content and shoot fresh weight also increased in JA-containing treatments. Taken together, foliar application of JA combined SA offers a promising strategy to enhance Se biofortification, reduce Cd accumulation, and promote biomass production in pak choi grown in Se-rich and high-Cd soils.

## 1 Introduction

Selenium (Se) is an essential trace element necessary to maintain the physiological health of humans and other animals (Sharma et al., 2010). About one billion people suffer from inadequate daily selenium intake across the world, posing a significant threat to public health (Combs, 2001). Because of its inherent toxicity and low bioavailability, inorganic Se is not suitable for direct human consumption; this means that enhanced dietary intake of organic selenium is necessary for Se supplementation (Stranges et al., 2007). Many crops are capable of assimilating inorganic selenium and converting it into organic forms that can be made available for consumption (Li et al., 2015). As a result, the biofortification of Se in crops represents a viable solution to address human selenium deficiency.

Biofortification of crops with Se is most effective in Se-rich soils (Se ≥ 0.4 mg/kg). However, Se-rich soils are often accompanied by elevated levels of cadmium (Cd) due to isomorphism, which can constrain the safe utilization of soils that are rich in Se (Zhang et al., 2019; Yang et al., 2019). Cd is a highly toxic trace heavy metal that is readily absorbed by plant roots and accumulates in edible tissues, posing serious health risks to animals and people (Zhang et al., 2014; Faizan et al., 2024a). Furthermore, high soil Se levels do not prevent Cd accumulation in crops (Yang et al., 2021), making it essential to simultaneously reduce Cd uptake while promoting Se enrichment. Exogenous Se application through foliar or soil treatments is commonly used to increase Se content and reduce Cd accumulation in crops (Huang et al., 2024). However, the effectiveness of Se in reducing Cd varies depending on multiple factors (Affholder et al., 2019; Yu et al., 2018), and excess Se can itself become toxic or even promote Cd uptake under certain conditions (Ismael et al., 2019). Additionally, exogenous Se application faces challenges including low plant utilization efficiency, limited Se availability, and environmental concerns (Sager, 2006; Zhang and Zhou, 2019). Soil amendments and microbial agents have also been explored to enhance availability of Se while immobilizing Cd (Yang et al., 2022), though potential ecological and health risks remain (Zhang et al., 2021).

Exogenous plant hormones are widely applied in crop production to enhance growth and regulate stress responses (Peleg and Blumwald, 2011; Alam et al., 2023). In addition to promoting growth, several hormones modulate ion transport processes that influence the uptake and translocation of both toxic and beneficial elements (Aftab and Hakeem, 2021). Jasmonic acid (JA) and salicylic acid (SA), for example, have been shown to reduce cadmium (Cd) accumulation in crops (Lei et al., 2020; Jia et al., 2021). Multiple hormones, including indole-3-acetic acid, abscisic acid, gibberellins, as well as JA, and SA, have also been reported to regulate selenium (Se) uptake and accumulation (Huan et al., 2021; Li et al., 2024; Guo et al., 2024; Chen et al., 2021; 2024). In this study, JA and SA were prioritized because of their well-established roles in sulfur assimilation pathways, which are directly linked to Se metabolism (Nazar et al., 2011; Tolu et al., 2022; Siddiqui et al., 2024). In addition, JA and SA contribute to Se bioaccumulation and help mitigate Se-induced stress in crops (Chen et al., 2021; 2024).

To date, most studies have focused on either high Cd or Se-rich soils, while the combined effects of exogenous hormone application in soils that are both Se-rich and high-Cd remain largely unexplored. Furthermore, previous research has focused on single-hormone treatments, which may be insufficient under complex stress conditions (Nazari et al., 2022). Integrated management strategies, including the use of hormone combinations, could potentially enhance the effectiveness of stress mitigation (Zeng et al., 2024; Faizan et al., 2021b). Notably, combined hormone applications often exhibit synergistic effects offering greater efficacy in addressing multifaceted environmental stresses compared to individual treatments (Tran and Pal, 2014), and may be more effective in reducing heavy metal accumulation (Modarresi et al., 2024; Faisal et al., 2024). Based on this, the hypothesis in this study is that the co-application of JA and SA will outperform individual hormone treatments by simultaneously promoting Se enrichment and Cd reduction.

Because vegetables are the primary source of Se in human diets (Rayman, 2012; Al-Othman et al., 2012), this study focused on pak choi (*Brassica chinensis*), an important vegetable crop with the potential to provide supplemental Se (Abdalla et al., 2020; Li et al., 2015). However, pak choi also has a higher propensity to accumulate Cd compared to other leafy vegetables (Wang et al., 2017b). This highlights the necessity to investigate the influence of exogenous plant hormones on both the Se uptake and Cd accumulation in Se-rich and high-Cd soils.

This study investigated the potential for plant hormone application to promote Se enrichment and Cd reduction in pak choi, as well as its potential underlying mechanisms. It did so by measuring the variation of Se and Cd accumulation, metal chelation compounds and physiological response of pak choi under Se-rich and high-Cd soils treated with and without plant hormones. Overall, this study will provide a basis for improving the efficient and safe use of Se-rich and high-Cd soils.

## 2 Materials and methods

### 2.1 Experimental materials and setup

Soil for the pot experiment was collected from a vegetable cultivation site in Gangbei district, Guigang city Guangxi China (109°45′57.32″E, 23°14′12.36″N). Soil surface samples (0–20 cm depth) were collected, homogenized, and air-dried after removing visible impurities and stones. The basic physicochemical properties of the soil were as follows: total selenium (Se), 0.27 mg·kg^-^¹; cadmium (Cd), 0.328 mg·kg^-^¹; total phosphorus (P), 0.95 g·kg^-^¹; total nitrogen (N), 2.26 g·kg^-^¹; total potassium (K), 3.0 g·kg^-^¹; organic matter, 36.2 g·kg^-^¹; and pH, 7.2.

Oval-shaped pots (37 cm long axis x 29 cm short axis x 9 cm height) were used, and each filled with 7.0 kg of air-dried soil. The air-dried soil was spiked with 1 mg·kg^-^¹ of Se^6+^ (Na_2_SeO_4_) and 1 mg·kg^-^¹ of Cd²^+^ (CdCl_2_·2.5H_2_O) to simulate Se-rich and Cd-contaminated soil; these values were derived from the Chinese National Standard for Soil Selenium Classification (GB/T 44971-2024), the Soil Environmental Quality Risk Control Standard for Soil Contamination of Agricultural Land (GB 15618-2018), and reported levels of Se and Cd concentrations in Se-enriched regions (Yu et al., 2020). To achieve the target Se level, analytical grade selenate (Na_2_SeO_4_) was applied to the dry soil using a plastic nebulizer while stirring continuously to ensure uniform distribution. The treated soil was then equilibrated for six months to allow available Se to stabilize. After application, soil was allowed to equilibrate for six months. Previous studies have shown that available Se reaches equilibrium within 33 to 56 days in acidic soils and within 109 days in neutral or alkaline soils (Wang et al., 2017a), confirming that the chosen six- month equilibration period was sufficient under the experimental conditions. Following Se equilibration, analytical grade cadmium chloride (CdCl_2_·2.5H_2_O) was applied using the same method to achieve the target concentration, with an equilibrium period of three months, based on evidence that Cd extractability tends to stabilize within 90 days after application (Zhang et al., 2018).

Seven treatments were established to assess the effects of different concentrations of JA or/and SA, as follows: (1) 0 μmol·L^-1^ plant hormones (no application of JA and SA, control) (hereafter, CK); (2) 20 μmol·L^-1^ JA (hereafter, JA20); (3) 50 μmol·L-1 JA (hereafter JA50); (4) 20 μmol·L-1 SA (hereafter SA20); (5) 50 μmol·L-1 SA (hereafter SA50); (6) 20 μmol·L^-1^ JA + 20 μmol·L^-1^ SA (hereafter JA20+SA20); (7) 50 μmol·L^-1^ JA + 50 μmol·L^-1^ SA (hereafter JA50+SA50). Each treatment included seventeen pots. In each pot, 15 pak choi seeds (‘Gui Tian Cai Xin No. 1’, bred by the Institute of Vegetable Research Institute, Guangxi Academy of Agricultural Sciences) were sown on February 20, 2024. After 15 days, seedlings were thinned to retain seven plants per pot, and 7 g of compound fertilizer was applied.

To prepare the hormone solutions, either JA or SA (Sigma-Aldrich, USA) was first dissolved in 7 mL of anhydrous ethanol and then diluted to a 1 mmol·L^-^¹ stock solution with distilled water. For the control treatment, an equal volume mixture of anhydrous ethanol and distilled water was used. When plants reached the five-leaf stage (March 14), 20 mL of the designated hormone solution was applied to each pot via hand sprayer. Treatments were repeated at six-day intervals for a total of three applications. Pots were randomly arranged within the growth facility and repositioned every three days to minimize microenvironmental variability.

### 2.2 Plant sample collection and physio-chemical parameters analysis

At harvest (April 7), plants were washed, separated into shoots and roots, and fresh weights were recorded. For all measurements, each biological replicate consisting of seven plants per pot. Selenium and cadmium accumulation and metal chelation compound analyses were performed using four replicates (four pots) per treatment. Physiological measurements were performed using three replicates (three pots) per treatment. Fresh weight measurements were performed using nine replicates (nine pots) per treatment.

Portions of each sample intended for analysis of metal chelation compounds, malondialdehyde (MDA), antioxidant enzymes, and photosynthetic pigments were immediately frozen and stored at -80°C.

Shoots and roots were oven-dried at 90°C for 30 minutes, followed by drying at 60°C until constant weight was achieved. Se content was determined using Atomic Fluorescence Spectrometry (AFS 8330, Jitian Instrument Co., Beijing). The Se translocation factor was calculated as the ratio of Se concentration in shoots to that in roots. Cadmium concentrations were determined using Graphite Furnace Atomic Absorption Spectrometry (55B+240Z Duo, Agilent Technologies, USA), and the Cd translocation factor was similarly calculated as the shoot-to-root concentration ratio. The content of MTs, non-protein thiols (NPTs) and PCs were measured using the respective plant enzyme-linked immunosorbent assay (ELISA) kits (Chongqing Bonoheng Biotechnology Company Limited, Chongqing, China). The content of GSH and MDA, and superoxide dismutase (SOD), peroxidase (POD), and catalase (CAT) activities of leaf were detected using extraction kits for each biochemical marker (Chongqing Bonoheng Biotechnology Company Limited, Chongqing, China) following the manufacturer’s instructions.

The content of photosynthetic pigment was determined using the ethanol-acetone extraction method (Lichtenthaler and Wellburn, 1983). Fresh leaf tissue (0.100 g) was homogenized and extracted with 10 mL of the extraction solution for 48 h in darkness. The absorbance of the extracts was measured spectrophotometrically at wavelengths of 440, 645, and 663 nm. The concentrations of chlorophyll a (Chl a), chlorophyll b (Chl b), and carotenoids (Car) were calculated according to standard equations. The total chlorophyll content (Chl a+b) and the ratio of total chlorophyll to carotenoid content (Total Chl (a+b)/Car) were also derived.

### 2.3 Data analysis

Treatment effects of JA and SA on Se and Cd content, metal chelation compounds content, and growth, physiological responses were analyzed using Analysis of variance (ANOVA). Duncan’s multiple comparison test was used to determine significant differences among treatments. Analyses were conducted using the SPSS 22 software; data on Se and Cd content, metal chelation compounds content were plotted using Origin Pro 8.5 software.

## 3 Results

### 3.1 Effect of treatment on Se and Cd in plant

**Figure 1:**
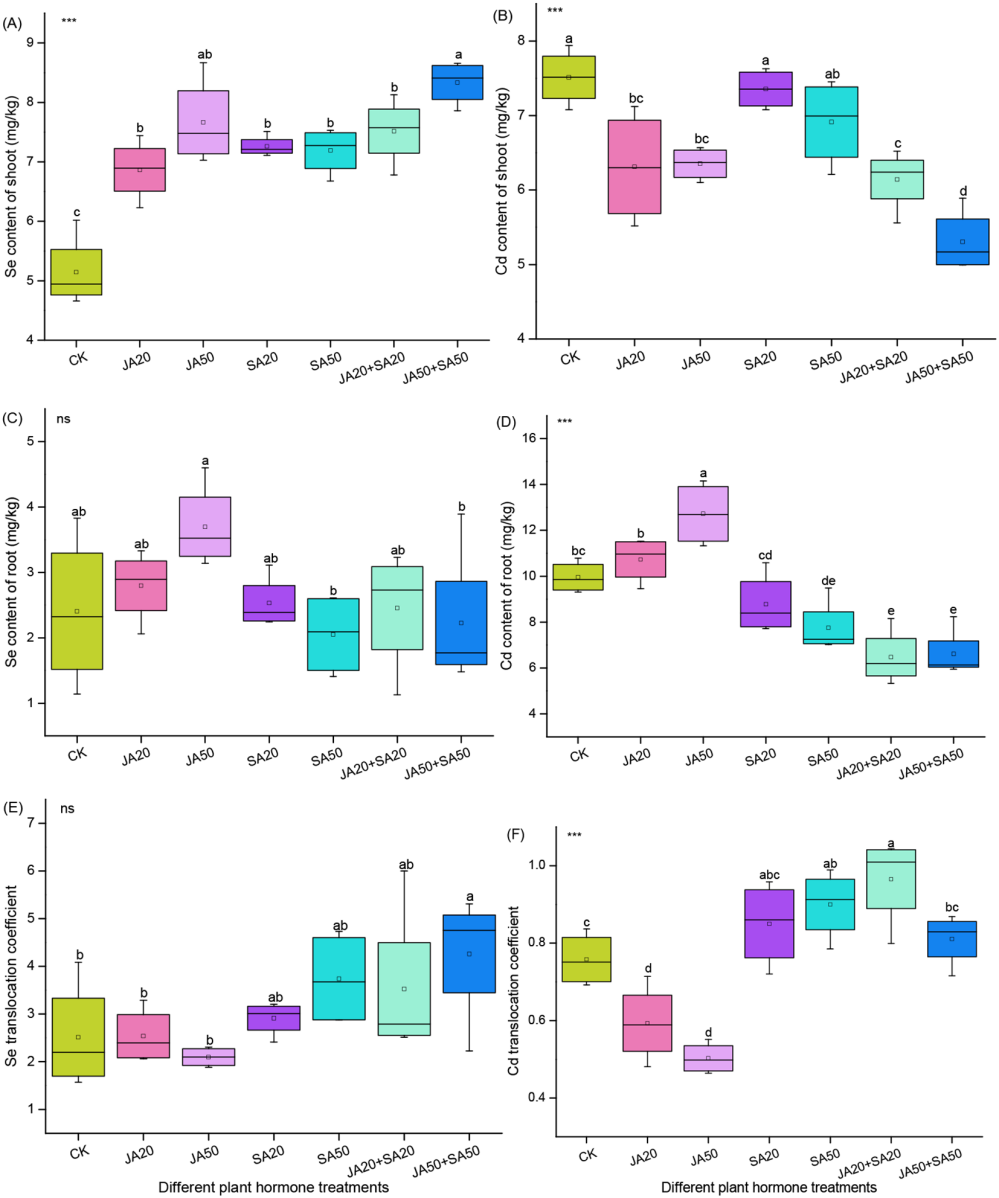
Effect of plant hormone treatment on the Se content of shoots **(A)** Cd content of shoots **(B)** Se content of roots **(C)** Cd content of roots **(D)**, as well as the Se translocation coefficient **(E)** and Cd translocation coefficient **(F)**. Data are mean ± SD (n=4). Asterisks (*) denote overall significant differences (ns, not significant; ***P **<** 0.001). Values with different lowercase letters in the same column indicate significant differences among treatments at the 0.05 level.

For Se content, all JA and SA treated groups had significantly higher levels in shoots compared to the control, with increases ranging from 33.7% to 62.3%; among these, the JA50+SA50 treatment group had the greatest increase (**Figure 1A**). There was no influence of exogenous plant hormones on selenium content in roots compared to the control (**Figure 1C**). The JA50+SA50 treatment led to a higher Se translocation coefficient, but there was no change in other hormone treatments (**Figure 1E**).

For Cd content, treatments with JA had lower Cd concentrations in shoots (11.7%-29.3%) compared to the control; the JA50+SA50 treatment had the biggest effect (**Figure 1B**). When SA was treated alone, there was no influence of Cd content in shoots (**Figure 1B**). When JA was treated alone, there was higher Cd content in roots (**Figure 1D**). All treatments containing SA had lower Cd content in roots (**Figure 1D**). When JA was added alone, the Cd translocation coefficient decreased, whereas treatments containing SA generally increased this parameter (**Figure 1F**).

### 3.2 Effect of treatment on metal chelation compounds

**Figure 2:**
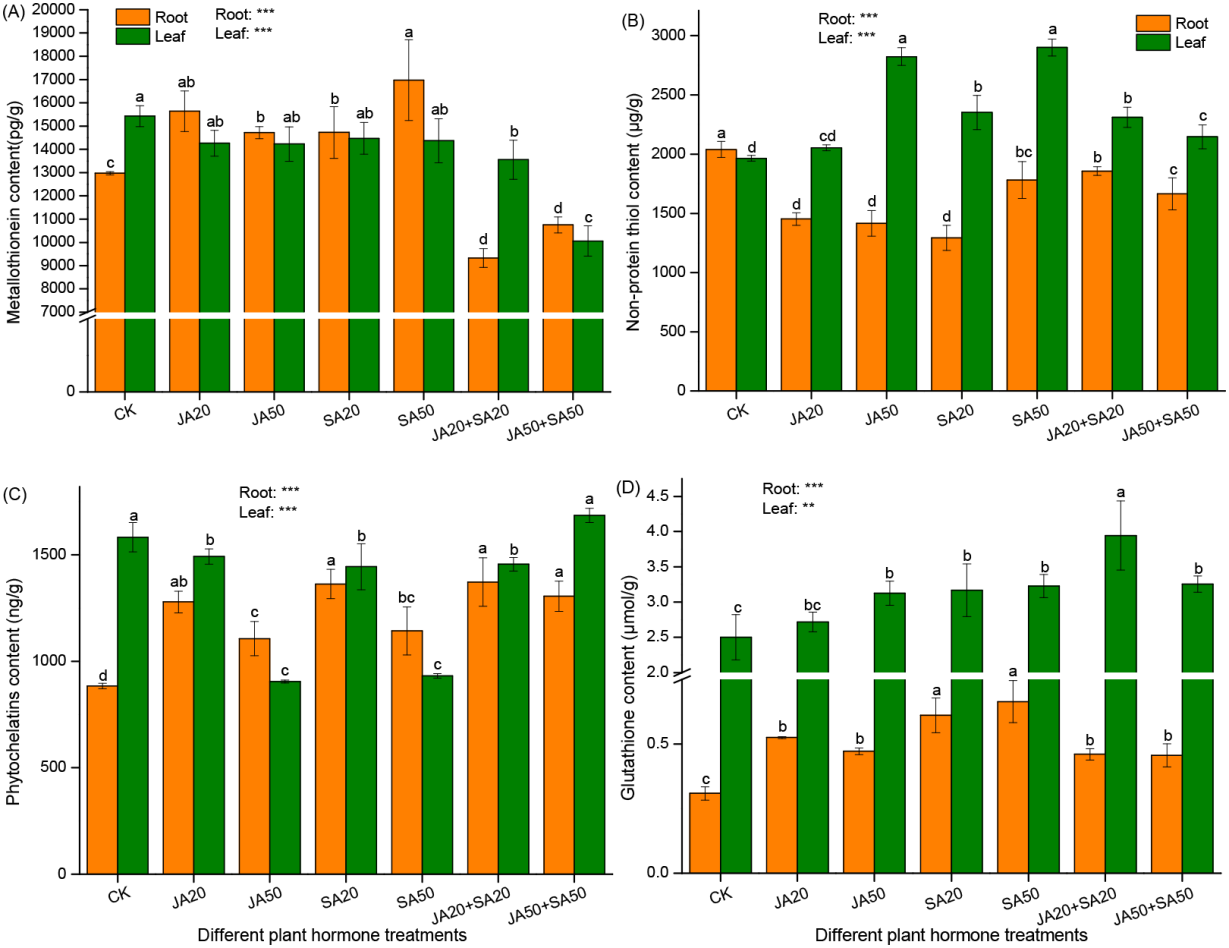
Effect of plant hormone treatment on root and leaf content of metallo-thioneins **(A)** non-protein thiol **(B)** phytochelatins **(C)**, and glutathione **(D)**. Data are mean ± SD (n=4). Asterisks (*) denote overall significant differences (**P **<** 0.01; ***P **<** 0.001). Values with different lowercase letters in the same column indicate significant differences among treatments at the 0.05 level.

In roots, treatments with only JA or SA led to higher content of metallo-thioneins (MTs) (**Figure 2A**), phytochelatins (PCs) (**Figure 2C**), and glutathione (GSH) (**Figure 2D**) compared to the control. On the other hand, these treatments led to notable reductions in non-protein thiol (NPTs) levels (**Figure 2B**). When both hormones were mixed, PCs and GSH increased, but MTs and NPTs decreased (**Figure 2**).

In leaves, plant hormone treatments generally promoted non-protein thiol (NPTs) (**Figure 2B**) and glutathione (GSH) (**Figure 2D**) (other than the JA20 treatment). On the other hand, all hormone treatments tended to lead to decreases in MTs (**Figure 2A**) and PCs (other than the JA50+SA50 treatment) concentrations (**Figure 2C**).

### 3.3 Effect of treatment on MDA content and antioxidant enzymatic activity

Hormone treatments led to significant variation in malondialdehyde (MDA) (**Table 1**). The JA20 treatment had lower MDA content in leaves, while there was no influence of the JA50 and SA20 treatments on MDA levels. Conversely, the mixed hormone treatments and SA50 led to higher MDA content (**Table 1**).

**Table 1:** Effect of plan hormone treatment on MDA content and antioxidant enzymatic activity of pak choi leaf.

Treatments	MDA content (nmol/g)	SOD activity (U/g)	POD activity (U/g)	CAT activity (nmol/min/g)
CK	122.81 ± 8.02c	36.61 ± 3.81d	7358.76 ± 492.98c	170.88 ± 14.62d
JA20	107.65 ± 11.95d	60.76 ± 5.47c	9943.60 ± 461.72b	280.82 ± 14.90bc
JA50	130.52 ± 11.50c	92.68 ± 11.00b	9164.27 ± 103.51b	322.12 ± 17.76a
SA20	136.52 ± 10.59c	57.00 ± 3.25c	10995.84 ± 600.01a	187.05 ± 15.45d
SA50	207.95 ± 18.85a	60.98 ± 0.34c	9811.09 ± 554.35b	263.38 ± 24.51c
JA20+SA20	159.78 ± 5.14b	63.23 ± 6.29c	8178.07 ± 545.00c	265.19 ± 22.23c
JA50+SA50	171.85 ± 15.31b	139.78 ± 14.12a	7707.23 ± 298.38c	314.39 ± 22.99ab

Data are mean ± SD (n=3). Values with different lowercase letters in the same column indicate significant differences among treatments at the 0.05 level.

All hormone treatments tended to enhance the activities of superoxide dismutase (SOD), peroxidase (POD), and catalase (CAT) compared to the control (**Table 1**). SOD was significantly enhanced by all treatments, while all hormone treatment other than SA20 increased CAT activity. While additions of both JA and SA alone led to higher POD activity, the addition of both hormones simultaneously had no influence on POD activity (**Table 1**).

### 3.4 Effect of treatment on the photosynthetic pigment content and fresh weight

Treatments with JA all had higher chlorophyll b content, total chlorophyll content, and the ratio of total chlorophyll to carotenoids; all but the JA20+SA20 treatment also had higher chlorophyll a content (**Table 2**). On the other hand, treatments with only SA had no influence on chlorophyll a content, but reduced chlorophyll b content, total chlorophyll content, and the ratio of total chlorophyll to carotenoids (**Table 2**).

**Table 2:** Effect of plant hormone treatment on the photosynthetic pigment content of pak choi plants.

Treatments	Chlorophyll a content (mg/g)	Chlorophyll b content (mg/g)	Total chlorophyll content (mg/g)	Carotenoid content (mg/g)	Total chlorophyll content/carotenoid content
CK	1.62 ± 0.08de	1.08 ± 0.09c	2.71 ± 0.01d	0.1621 ± 0.0052bc	16.71 ± 0.54c
JA20	2.35 ± 0.08a	1.41 ± 0.07b	3.76 ± 0.06b	0.1783 ± 0.0019a	21.06 ± 0.41b
JA50	1.89 ± 0.09c	1.35 ± 0.13b	3.23 ± 0.14c	0.1582 ± 0.0058cd	20.48 ± 1.61b
SA20	1.55 ± 0.08e	0.65 ± 0.11d	2.20 ± 0.04f	0.1691 ± 0.0066ab	13.01 ± 0.71d
SA50	1.66 ± 0.02de	0.74 ± 0.10d	2.40 ± 0.09e	0.1713 ± 0.0053a	14.04 ± 0.93d
JA20+SA20	1.74 ± 0.14cd	1.40 ± 0.08b	3.14 ± 0.06c	0.1518 ± 0.0056d	20.72 ± 1.14b
JA50+SA50	2.09 ± 0.14b	1.96 ± 0.09a	4.05 ± 0.19a	0.1513 ± 0.0016d	26.75 ± 1.51a

Data are mean ± SD (n=3). Values with different lowercase letters in the same column indicate significant differences among treatments at the 0.05 level.

**Figure 3:**
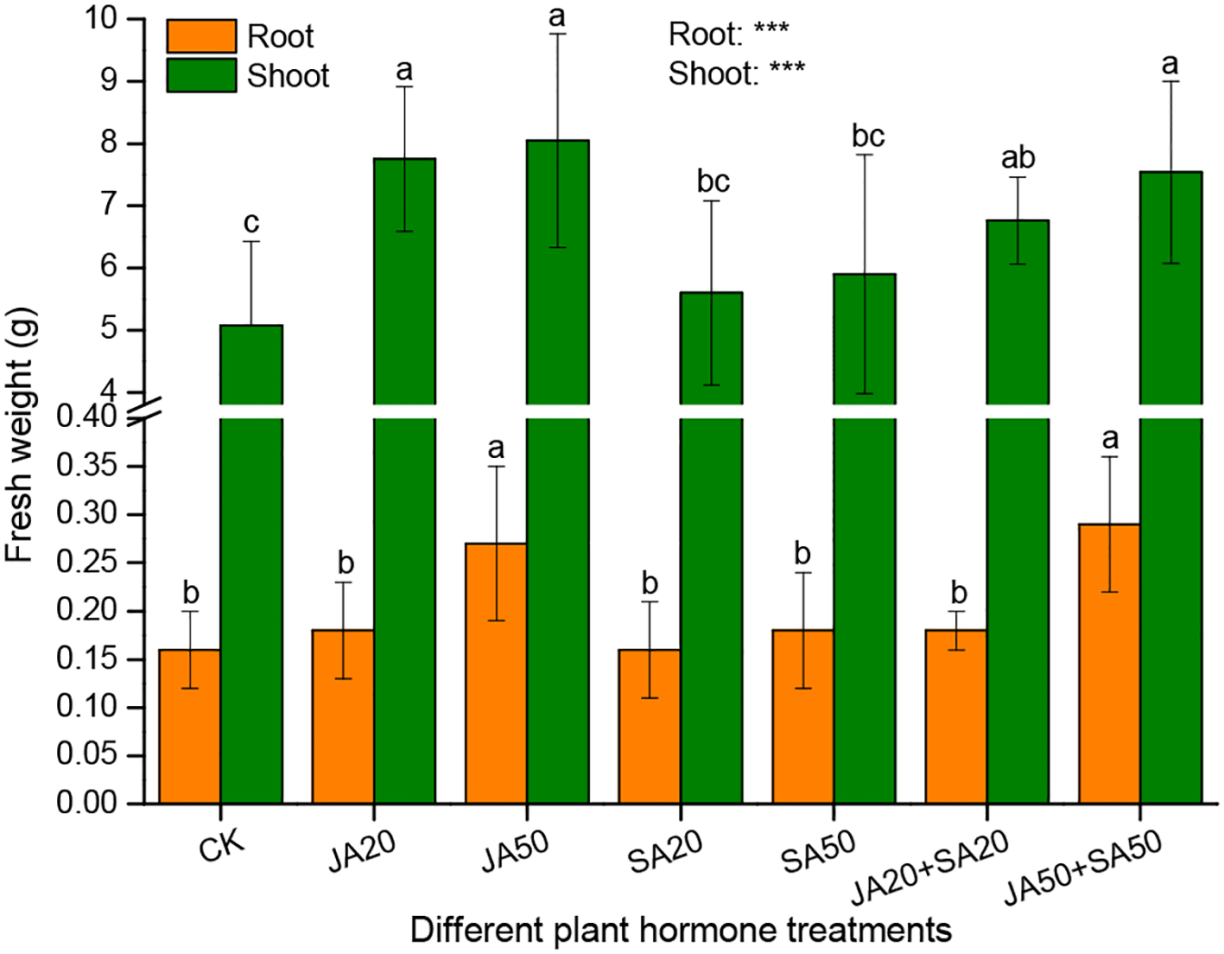
Effect of plant hormone treatment on plant fresh weight. Data are mean ± SD (n=9). Asterisks (*) denote overall significant differences (***P **<** 0.001). Values with different lowercase letters in the same column indicate significant differences among treatments at the 0.05 level.

The fresh weight of shoots was higher in JA-containing treatments compared to controls and those with SA alone (**Figure 3**). Likewise, the fresh weight of roots was higher in the JA50 and JA50+SA50 treatments (**Figure 3**). However, there was no influence on treatments with SA alone on shoot or root fresh weight (**Figure 3**).

## 4 Discussion

Plant hormones play a pivotal role in the uptake of ions and their distribution in plants (Starck and Kozińska, 1980). Several exogenous plant hormones have been shown to enhance the uptake and accumulation of selenium (Se) in crops, including indole-3-acetic acid (IAA), diethyl aminoethyl hexanoate (DA-6), JA, SA, abscisic acid (ABA), gibberellin (GA_3_), and melatonin (Liu et al., 2021; Li et al., 2022b; Huan et al., 2021; Li et al., 2024; Guo et al., 2024; Chen et al., 2024). Among these, JA, SA, ABA, and GA_3_ have been shown to enhance Se accumulation, particularly under Se stress (Li et al., 2022b, 2024; Guo et al., 2024; Chen et al., 2021; 2024).

This study showed that the application of JA, SA, and their combination all led to increased Se content of pak choi grown in Se-rich and high-Cd soils; the JA50+SA50 mixture had the greatest effect. The ability of exogenous plant hormones to promote Se uptake overcomes the bottleneck of plant Se tolerance and holds great potential for enhancing organic Se production in crops. Although the precise molecular mechanisms remain unclear, several potential pathways have been proposed. First, plant hormones may promote overall plant growth, increasing sink demand for Se and thereby facilitating its assimilation (Guo et al., 2024; Li et al., 2024; Chen et al., 2021; 2024). Second, given the chemical similarity between S and Se, plant hormones may regulate Se uptake by modulating sulfur transport and metabolism. Because Se assimilation primarily occurs via the S metabolic pathway (Tolu et al., 2022), hormonal regulation of S metabolism can influence Se uptake. For instance, ABA can stimulate the biosynthesis and signaling of jasmonic acid (JA) and salicylic acid (SA), upregulate genes encoding sulfate and phosphate transporter proteins, and enhance the reduction of inorganic S/Se to organic forms. This leads to increased assimilation and biosynthesis of S/Se-containing compounds (Wang et al., 2024). Exogenous JA and SA may mimic endogenous hormone signaling, further enhancing sulfur metabolism and facilitating Se uptake and translocation (Siddiqui et al., 2024; Nazar et al., 2011). Finally, plant hormones may induce specific root responses and alter rhizosphere metabolism, indirectly increasing the bioavailability of Se in the soil (Chen et al., 2024).

In this study, JA treatment reduced the root-to-shoot Cd translocation coefficient while increasing Cd accumulation in roots, suggesting that JA promotes Cd sequestration in roots and limits translocation to aboveground tissues. Similar results have been reported in *Arabidopsis thaliana*, where JA treatment reduced shoot Cd accumulation through downregulation of key Cd transporters, including AtIRT1, AtHMA2, and AtHMA4 (Lei et al., 2020). JA has also been shown to enhance Cd compartmentalization within root cell walls by promoting Cd binding to chelated-soluble pectin and reducing Cd influx into protoplasts (Li et al., 2022a), thereby increasing Cd retention in root tissues. In addition to JA, salicylic acid (SA) has been reported to influence Cd uptake and transporter gene expression (Jia et al., 2021), although its independent effect appeared limited under the current conditions, consistent with concentration-dependent efficacy thresholds (Tang et al., 2023). The internal redistribution of Cd toward roots minimizes accumulation in photosynthetic tissues, reduces phytotoxicity, and supports biomass production, which is critical for plant growth and long-term phytoremediation potential (Li et al., 2023). This sequestration is particularly advantageous for limiting Cd entry into the edible tissues of pak choi.

The combined JA+SA treatment further enhanced Cd mitigation compared to single-hormone applications. This may reflect hormone crosstalk influencing rhizosphere processes, including the production of secondary metabolites that alter metal chelation, solubility, and mobility (Hou and Tsuda, 2022). Similar synergistic effects have been observed in *Alyssum inflatum* exposed to nickel, where JA+SA co-application more effectively reduced metal accumulation than either hormone alone (Modarresi et al., 2024). In our study, this hormonal interaction may have reduced Cd bioavailability in the soil, limiting uptake and accumulation (Shi et al., 2024a). The synergistic regulation of rhizosphere metabolism and root physiology by JA and SA likely contributes to the greater effectiveness of the combined treatment in mitigating Cd stress.

Metal chelation compounds such as phytochelatins (PCs), glutathione (GSH), and metallothioneins (MTs), along with non-protein thiols (NPTs) play important roles in Cd detoxification by binding intracellular Cd and reducing its toxicity (Liu et al., 2012). In this study, single-hormone treatments increased concentrations of root-associated PCs, GSH, and MTs, suggesting that exogenous JA and SA enhance Cd tolerance by promoting the synthesis of sulfur-containing chelators that facilitate Cd sequestration in roots. The observed reduction in NPTs may reflect their consumption as precursors during GSH and PC biosynthesis (Seth et al., 2008). These hormone-induced changes appear linked to transcriptional regulation of sulfur assimilation genes (Shi et al., 2024a) and activation of enzymes such as glutathione reductase and phytochelatin synthase (Szalai et al., 2013). Interestingly, mixed JA+SA treatments resulted in lower MT concentrations in both roots and shoots, likely due to reduced Cd accumulation in these tissues and thus lower cellular demand for MT synthesis. Indeed, Cd concentrations in both roots and shoots were positively correlated with MT content (**Supplementary Material**), indicating a close relationship between intracellular Cd levels and MT response.

In addition to this Cd-dependent regulation, hormone crosstalk may also contribute to the observed changes in MT synthesis. SA can suppress JA signaling downstream of the JA receptor COI1 (Van der Does et al., 2013), inhibiting JA-responsive genes, including those involved in thiol compound synthesis. Furthermore, SA induces expression of the WRKY70 transcription factor, which acts as a key regulatory switch between JA and SA pathways, repressing JA-dependent defenses while activating SA-mediated responses (Li et al., 2004). Thus, in the combined JA+SA treatment, SA may have attenuated MT biosynthesis by modulating JA-responsive transcriptional networks.

Under normal conditions, excessive levels of Se or Cd can independently induce toxic effects on plants (Chen et al., 2024; Ismael et al., 2019; Elhakem et al., 2025). However, in Se-rich and high-Cd soils, the interactions between these elements is modulated by the bioavailable Se/Cd molar ratio, with critical thresholds determining whether the interaction is synergistic or antagonistic (Yang et al., 2022). This study reveals that different hormone treatments produced variable effects on the malondialdehyde (MDA) content in pak choi leaves—a key indicator of lipid peroxidation and oxidative stress. As such, high MDA content is related to oxidative stress (Faizan et al., 2021a). Notably the JA20 treatment reduced MDA levels, indicating enhanced protection against oxidative damage. In contrast, treatments with both hormone treatments, as well as SA50, elevated MDA content, suggesting increased cellular peroxidation. These findings are consistent with the concept of the “dual effects” of plant hormones, which may either promote or inhibit physiological stress depending on context. Indeed, the efficacy of exogenous hormone treatments is highly dependent on both the applied concentration and the severity of environmental stress (Chen et al., 2021). Under low- or no-stress conditions, excessive use of plant hormones may induce phytotoxicity effects rather than confer benefits (Hayat et al., 2013; Agnihotri and Seth, 2020).

All plant hormone treatments increased the activities of key antioxidant enzymes—SOD, CAT, and POD—which play central roles in scavenging reactive oxygen species (ROS) and reducing oxidative damage (Li and Ma, 2021). The enhanced antioxidant enzyme activity likely contributed to the observed decrease in malondialdehyde (MDA) levels, particularly under JA20 treatment, indicating reduced lipid peroxidation and membrane damage. This upregulation of enzymatic defenses appears to be mediated, at least in part, by JA- and SA-dependent activation of transcriptional regulators that modulate antioxidant gene expression, ultimately strengthening systemic acquired resistance and improving tolerance to Cd-induced oxidative stress (Shi et al., 2024a; b). These coordinated cellular defense responses collectively enhance the resilience of pak choi under Cd exposure and hormonal modulation.

Chlorophyll (a, b) levels typically decline under heavy metal stress due to chloroplast damage in the leaf mesophyll (Faizan et al., 2024b). In this study, treatment with JA or combined JA+SA increased chlorophyll a and b levels, suggesting enhanced light absorption photosynthetic efficiency. This enhancement may result from JA-mediated suppression of chlorophyll degradation (Su et al., 2021) and activation of key genes involved in the photosynthetic electron transport chain (Simkin et al., 2022). JA also regulates the synthesis of photosynthetic pigments by regulating jasmonate-ZIM-domain (JAZ) proteins, which interact with transcription factors to control gene expression related to growth and stress responses (Shi et al., 2024a). Improved photosynthetic performance likely supports higher biomass production (Zhao et al., 2019), increasing sink demand for selenium (Se) in developing tissues, and thereby promoting Se uptake and accumulation. This relationship also provides a potential ‘proof-of-concept’ for using JA-based hormonal treatments to enhance Se biofortification in crops grown in Se-rich but high-Cd soil. Stable biomass production combined with increased Se uptake is critical for improving the production efficiency of crop Se enrichment. Given Se’s essential role as a micronutrient for animals and humans—particularly in Se-deficient regions where related health issues are prevalent (Fairweather-Tait et al., 2011)—such strategies have significant implications for public health.

## 5 Conclusion

This study showed that under Se-rich and high-Cd soil conditions, exogenous application of plant hormones significantly increased Se accumulation in the shoot tissues of pak choi, while concurrently reducing Cd levels. Among the treatments, the combined foliar application of 50 μmol·L^-^¹ jasmonic acid (JA) and 50 μmol·L^-^¹ salicylic acid (SA) (JA50+SA50) resulted in the highest shoot Se content and the lowest Cd accumulation. Mechanistically, these effects appear to involve hormone-regulated modulation of root Cd sequestration, antioxidant defenses, sulfur-containing metal chelators (phytochelatins and glutathione), chlorophyll metabolism, and rhizosphere-mediated changes in metal bioavailability. The integration of these physiological responses not only improved Se/Cd ratios in edible tissues but also supported overall plant growth under combined metal stress. These findings provide proof-of-concept for the potential use of JA and SA co-application as a strategy to improve Se biofortification while mitigating Cd risk in crops grown on Se-rich but high-Cd soils. Nevertheless, further research, including large-scale field trials assessing agronomic feasibility, economic cost, and crop quality under variable environmental conditions, is essential to validate the practical applicability of this approach for sustainable food production and public health benefit.

## Data Availability

The original contributions presented in the study are included in the article/supplementary material. Further inquiries can be directed to the corresponding authors.
