# Supplementary material for: Exogenous jasmonic acid and salicylic acid enhance selenium uptake and mitigate cadmium accumulation in pak choi (Brassica chinensis L.) grown in selenium-rich, high-cadmium soil
Source: Front Plant Sci. 2025 Aug 19;16:1619522. doi: 10.3389/fpls.2025.1619522 (PMC12401708; doi:10.3389/fpls.2025.1619522)
Supplement: Supplementary file 1 [file Table1.docx]

**Supplementary material**

**Article title**: Exogenous jasmonic and salicylic acid enhance selenium uptake and mitigate cadmium accumulation in pak choi (*Brassica chinensis* L.) grown in selenium-rich, high-cadmium soil

**Journal name**: Frontiers in Plant Science

**Author names**: Jin-Ping Chen^1,2^ · Ying Xing^1,2^ · Qing Liao^1,2^ · Li-Ping Pan^1,2^ · Cheng-Cheng Zeng^1,2^ · Jie Qin^3*^ · Yong-Xian Liu^1,2*^

**Affiliation:**

1 Agricultural Resources and Environment Research Institute, Guangxi Academy of Agricultural Sciences, Nanning, China, 2 Guangxi Key Laboratory of Arable Land Conservation, Nanning, China,

3 Flower Research Institute, Guangxi Academy of Agricultural Sciences, Nanning, China

**Corresponding author:**

Jie Qin, E-mail address: 55459892@qq.com

Yong-Xian Liu, E-mail address: liuyx27@163.com

**Table 1**. Pearson correlation coefficients among the Cd/Se content and metal chelation compounds content of pak choi exposed to different treatments

|  | MTs content of leaf | MTs content of root | NPTs content of leaf | NPTs content of root | PCs content of leaf | PCs content of root | GSH content of leaf | GSH content of root |
| --- | --- | --- | --- | --- | --- | --- | --- | --- |
| Cd content of shoot | 0.694^**^ | 0.414^*^ | 0.077 | 0.159 | -0.132 | -0.375^*^ | -0.279 | 0.043 |
| Cd content of root | 0.488^**^ | 0.505^**^ | 0.104 | -0.372 | -0.372 | -0.383 | -0.466^*^ | -0.163 |
| Se content of shoot | -0.679^**^ | -0.175 | 0.375 | -0.444^*^ | -0.142 | 0.534^**^ | 0.586^**^ | 0.376^*^ |
| Se content of root | 0.150 | 0.102 | 0.166 | -0.284 | -0.260 | -0.038 | -0.140 | -0.128 |

Correlation was analyzed by the Pearson correlation coefficient. * Represents a significant difference (P < 0.05), ** represents an extremely significant difference (P < 0.01).
